# Accuracy of Support-Vector Machines for Diagnosis of Alzheimer's Disease, Using Volume of Brain Obtained by Structural MRI at Siriraj Hospital

**DOI:** 10.3389/fneur.2021.640696

**Published:** 2021-05-10

**Authors:** Yudthaphon Vichianin, Anutr Khummongkol, Pipat Chiewvit, Atthapon Raksthaput, Sunisa Chaichanettee, Nuttapol Aoonkaew, Vorapun Senanarong

**Affiliations:** ^1^Department of Radiological Technology, Faculty of Medical Technology, Mahidol University, Bangkok, Thailand; ^2^Division of Neurology, Department of Medicine, Faculty of Medicine Siriraj Hospital, Mahidol University, Bangkok, Thailand; ^3^Department of Radiology, Faculty of Medicine Siriraj Hospital, Mahidol University, Bangkok, Thailand

**Keywords:** Alzheimer disease, support vector machine, machine learning, volumetric MRI, Thailand

## Abstract

**Background:** The determination of brain volumes using visual ratings is associated with an inherently low accuracy for the diagnosis of Alzheimer's disease (AD). A support-vector machine (SVM) is one of the machine learning techniques, which may be utilized as a classifier for various classification problems. This study exploratorily investigated the accuracy of SVM classification models for AD subjects using brain volume and various clinical data as features.

**Methods:** The study was designed as a retrospective chart review. A total of 201 eligible subjects were recruited from the Memory Clinic at Siriraj Hospital, Thailand. Eighteen cases were excluded due to incomplete MRI data. Subjects were randomly assigned to a training group (AD = 46, normal = 46) and testing group (AD = 45, normal = 46) for SVM modeling and validation, respectively. The results in terms of accuracy and a receiver operating characteristic curve analysis are reported.

**Results:** The highest accuracy for brain volumetry (62.64%) was found using the hippocampus as a single feature. A combination of clinical parameters as features provided accuracy ranging between 83 and 90%. However, a combination of brain volumetry and clinical parameters as features to the SVM models did not improve the accuracy of the result.

**Conclusions:** In our study, the use of brain volumetry as SVM features provided low classification accuracy with the highest accuracy of 62.64% using the hippocampus volume alone. In contrast, the use of clinical parameters [Thai mental state examination score, controlled oral word association tests (animals; and letters K, S, and P), learning memory, clock-drawing test, and construction-praxis] as features for SVM models provided good accuracy between 83 and 90%.

## Introduction

Alzheimer's disease (AD) is a common condition that is diagnosed in ~5–7% of the general population (1). Current treatment can improve the quality of life of both Alzheimer's patients and their relatives and caregivers (2). Consequently, a tool that provides high sensitivity and specificity is needed for the accurate diagnosis of this disease.

The current diagnostic tool for AD is the use of clinical criteria, such as DSM-V (3). Despite imaging studies not being included in such criteria, many clinicians have noticed that the size of the brain volume obtained from structural imaging appears to be associated with AD to some extent. Although visual rating of the brain volume is generally used to guide a diagnosis of AD, its interpretation differs vastly among clinicians, and the method lacks specificity and sensitivity (4). It would be beneficial if there was a reliable tool that could interpret imaging results accurately and consistently.

**Figure 1:**
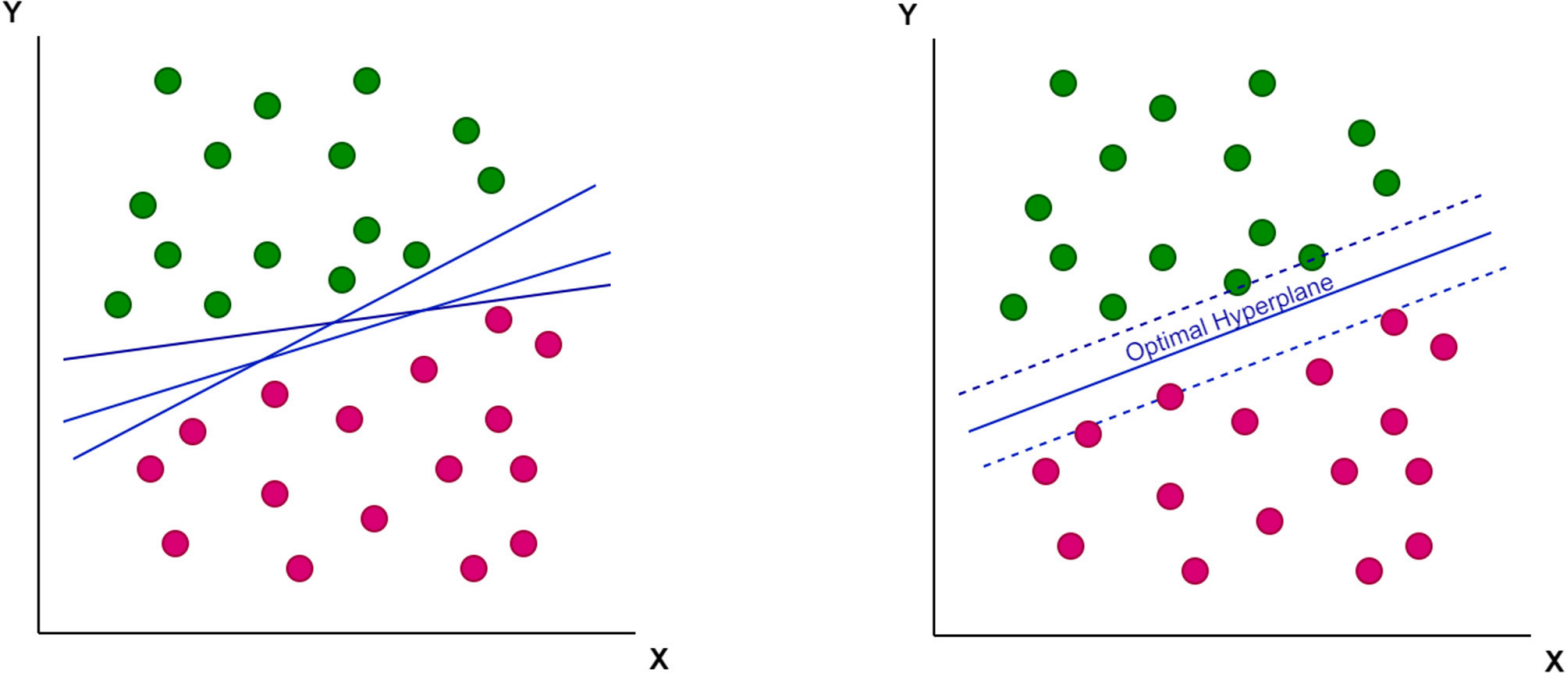
The concept of optimal hyperplane to properly classify the data into two groups.

**Figure 2:**
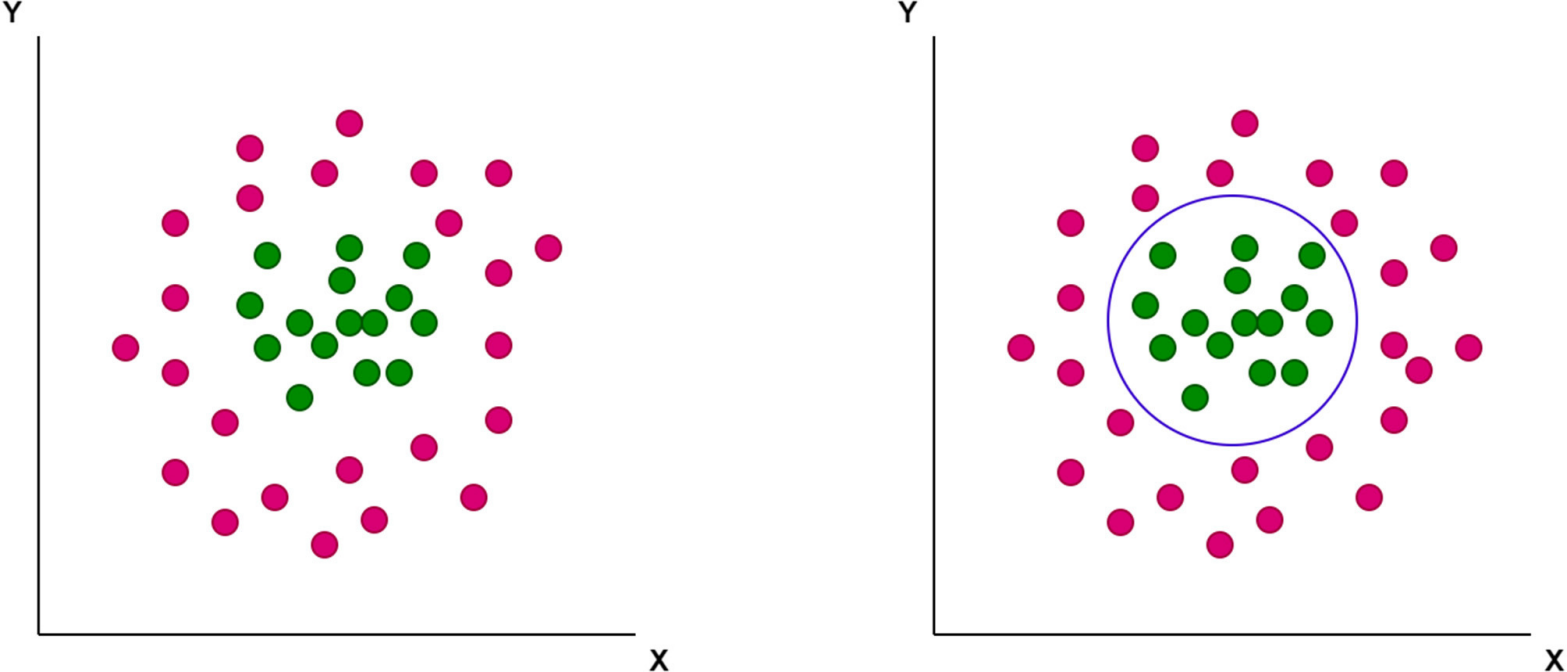
The non-linear optimal hyperplane, which support-vector machine (SVM) can provide as a classification tool.

A support-vector machine (SVM), a mathematical function, is designed to classify complex data. This function has the ability to learn the distribution of data and provide a proper classification line (or optimal hyperplane) that is not restricted to a linear fashion (Figures 1, 2).

Several studies have investigated SVM as a diagnostic tool for AD, and a number have shown good levels of accuracy (5–8). However, those results were based on international imaging data obtained from the Alzheimer's Disease Neuroimaging Initiative, which included a different population from the Thai cohort used in the current study. Hence, this study focused on the accuracy of SVM as a diagnostic tool for the Thai population. Moreover, we investigated clinical data in order to determine if it is possible to further increase the accuracy of SVM.

## Materials and Methods

### Study Design

This study was a retrospective, chart review and was approved by the Siriraj Institutional Review Board. Everybody had signed informed consent as part of data registration at the Memory Clinic at Siriraj Hospital. The subjects were recruited by clinical coordinators from the Memory Clinic at Siriraj Hospital. Some normal subjects had brain magnetic resonance imaging scan, which was supported by the Thailand Research Fund. All were living in the community. Their identities (name and identification number) were hidden from the staff.

For the present study, the inclusion criteria consisted of the following: aged 60 years or older, a diagnosis of AD, in accordance with the (9) criteria, and the performance of an MRI brain scan within 1 year of the AD diagnosis. The exclusion criteria were having an untreated psychiatric condition; having a history of stroke (either ischemic or hemorrhagic), CNS infection, drug abuse, epilepsy, severe head trauma, and/or repetitive head trauma; and having been diagnosed with another type of dementia (such as frontotemporal dementia, Parkinson's disease with dementia, Lewy body dementia, vascular dementia, and other secondary dementia).

### Subjects

A total number of 201 subjects in this study consists of 101 AD subjects recruited from the memory clinic at Siriraj Hospital, and 100 normal subjects were enlisted from volunteers from the dementia and disability project (10) and caregivers from the memory clinic. These normal controls did not meet the criteria of dementia or mild cognitive impairment. Eighteen subjects were removed due to incomplete MRI data necessary for further analysis.

**Figure 3:**
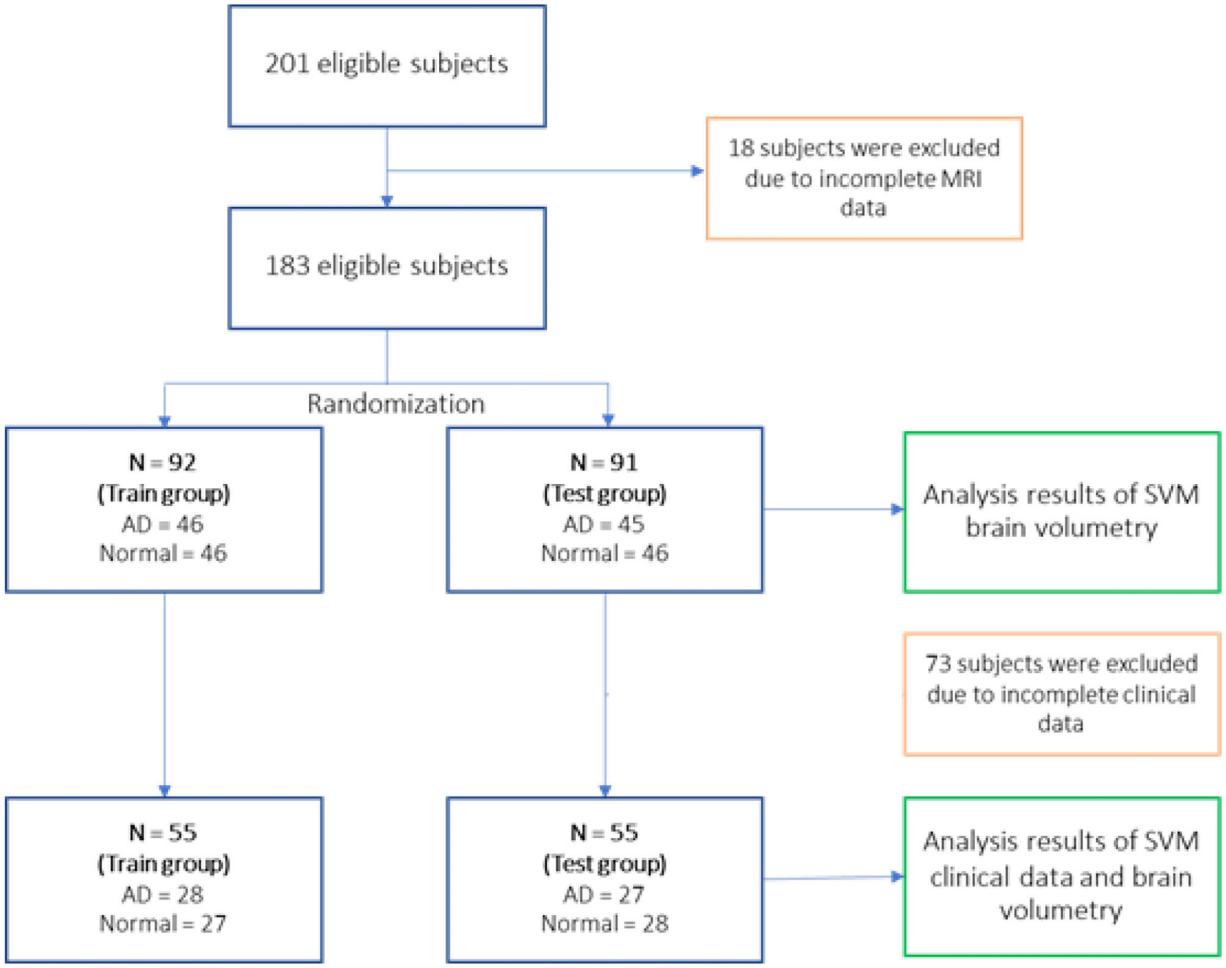
Actual study process after the exclusion of subjects.

Eligible 183 subjects then were randomly divided into the training group (*N* = 92 with AD = 46, normal = 46) and testing group (*N* = 91 with AD = 45, normal = 46) for the SVM study using brain volumetry alone. However, due to the subjects' incomplete clinical data, 73 subjects were removed resulting to 55 subjects for the training group (AD = 28, normal = 27) and 55 subjects for the testing group (AD = 27, normal = 28) of the SVM study using a combination of clinical data and brain volumetry. The randomization technique was used for assigning subjects into groups in order to minimize selection bias and ensures against the accidental uncontrolled bias (11) (Figure 3).

### Clinical Data

The Thai mental state examination (TMSE) (12) is a cognitive assessment modified from the Mini mental status examination. Thai mental state examination consists of seven subdomains: orientation, immediate memory (registration), calculation, attention, language, picture copy, and recalled memory. The total score is 30. The score of <24 is suggestive of having dementia. The higher the TMSE score, the better the cognitive function.

The Neuropsychiatric Inventory (NPI-Q) (13) is used to evaluate neuropsychiatric symptoms of all subjects. The NPI-Q is an informant-based instrument that measures the presence and severity of 12 neuropsychiatric symptoms in patients with dementia and normal controls, and to measure informant distress according to individual neuropsychiatric symptom of patients with dementia.

The cognitive data: the examination scores of TMSE and other neuropsychological assessments, namely, Logical Memory (LM), Controlled Oral Word Association Test—animals (COWA-animals), Controlled Oral Word Association Test—letters K, S, P (COWA-KSP), Clock drawing, and Construction—praxis were used as clinical parameters for further SVM model developments. Neuropsychological evaluation is part of clinical criteria to determine if subjects have dementia.

### MRI Data

Whole brain MR (3-Tesla) T1-weighted axial 3D Turbo fast field echo (3D TFE) MR structural images were retrieved from the hospital database [voxel size = 1 × 1 × 1 mm, repetition time (TR) = 7.7 ms, echo time (TE) = 3.6 ms, flip angles = 80, TFE factor = 144, FOV = 230 × 290 mm, matrix = 232 × 288, slice thickness = 1 mm, NSA = 1]. The identities of the patients were subsequently anonymized by the researchers.

### Analysis

The MR images were processed using FreeSurfer to seek for brain volumes (14). Brain MR image analysis was performed using the Freesurfer software suite to extract the brain cortical and subcortical brain volume (15).

Because the individuals had different cranial volumes, all specific regions of the brain volume were calculated relative to the whole brain volume, using the following normalization formula:

Specific volume of the brain × 100/intracranial volume

The specific volumes used to develop SVM models comprised of both sides of the hippocampus, nucleus accumbens, amygdala, caudate, thalamus, total white matter volume, total gray matter volume, ventricle volume, and a combination of those values.

The clinical data parameters used for SVM models in this study were the score from various tests including the TMSE, LM, COWA—animals, COWA-KSP, Clock, Construction—praxis, NPI 4, NPI 5, NPI 7, NPI 9, and NPI 12.

The WEKA software suite (available at https://www.cs.waikato.ac.nz/ml/weka/) was utilized in this study for SVM classification. Radial basis function was used to seek for the highest accuracy as suggested in a prior study (15). The SVM models then were trained and tested using the data from the training and testing groups (11). The leave-one-out validation technique was applied due to the small data set. The C and gamma values were adjusted to maximize the accuracy and area under the curve of the ROC for all SVM models as shown in **Table 3**.

The demographic data were analyzed and compared by descriptive statistics, using the unpaired *t*-test or Chi-square-test, according to each variable's type. We considered any *p*-value < 0.05 as statistically significant. The results were reported as true positive rate (TP rate), false positive rate (FP rate), accuracy, and the area under the curve (AUC) in a receiver operating characteristic curve (ROC) analysis.

## Results

An analysis of the demographic data (Table 1) revealed an average age of 73.09 years (SD = 7.28) for the AD group, and a lower average of 69.72 years for the normal controlled group, with a significant difference between those averages. Education levels and baseline TMSE scores also differed between the two groups. Even if there are some baseline differences, the randomization method we used with SVM and the leave one out method have something to help to compromise these baseline differences.

**Table 1:** Descriptive statistic of demographic data [mean (SD)].

	**AD group (*N* = 91)**	**Normal controlled group** **(*N* = 93)**	***P*-value**
Age	73.09 (7.28)	69.72 (6.12)	0.001
**Sex**			
Male (*N*)	32	40	0.234
Female (*N*)	59	53	
Education (years)	7.40 (5.63)	9.63 (5.67)	0.008
TMSE	21.26 (5.19)	26.83 (2.32)	<0.001
BMI	24.47 (5.00)	24.32 (3.56)	0.906
MAP mmHg	97.38 (13.47)	98.95 (11.52)	0.450
Smoking (*N*)	7	7	0.402
Hypertension (*N*)	31	36	0.942
DM (*N*)	19	13	0.119

*AD, Alzheimer's disease; TMSE, Thai Mental State Examination; BMI, body mass index; MAP, mean arterial pressure; DM, diabetes mellitus*.

As to the descriptive statistical analysis of the brain volumetry of the two groups, only the ventricle volume demonstrated a significant difference, being higher for the AD group (Table 2). Other parameters showed no significant differences.

**Table 2:** Descriptive statistics of brain volumetry.

**Volumes**	**AD group (*N* = 91)**	**Normal controlled group (*N* = 93)**	***p*-value**
Ventricle	5.43 (2.48)	4.46 (2.01)	0.004^*^
Gray matter	48.36 (10.99)	50.07 (5.39)	0.181
White matter	52.71 (7.99)	50.34 (5.76)	0.022^*^
Nucleus accumbens	0.14 (0.24)	0.16 (0.29)	0.523
Hippocampus	0.34 (0.19)	0.41 (0.22)	0.038^*^
Amygdala	0.19 (0.18)	0.21 (0.23)	0.431
Caudate	0.31 (0.19)	0.35 (0.19)	0.154
Thalamus	0.56 (0.37)	0.62 (0.33)	0.246

*The values below are calculated from “specific brain volume × 100/intracranial volume”*.

* *p < 0.05 = statistical significant; AD, Alzheimer's disease*.

From the SVM modeling performance analysis results in Table 3, two models with equally highest accuracy (90.74%) were the model of clinical parameters (TMSE, LM, COWA—animals, COWA-KSP, Clock drawing, and Construction—praxis scores) (item 16 in Table 3) and the model of all brain volumetry combined with the scores of TMSE, LM, COWA—animals, COWA-KSP, Clock drawing, and Construction—praxis scores (Table 3, item 18).

**Table 3:** Results of support vector machine (SVM).

	**Brain volumetry**								**Clinical parameters**											**SVM parameters**											
	**Hippo**	**Accumbent**	**Amygdala**	**Caudate**	**Thalamus**	**White.M**	**Gray.M**	**Ventricle**	**TMSE**	**LM**	**COWA-animals**	**COWA-KSP**	**Clock**	**Conpraxis**	**NPI4**	**NPI5**	**NPI7**	**NPI9**	**NPI 1–12**	**Setting**		**Trained set**					**Tested set**				
																				***C***	**Gamma**	***N***	**TP rate**	**FP rate**	**Accuracy**	**ROC**	***N***	**TP rate**	**FP rate**	**Accuracy**	**ROC**
1	x																			10	100	92	0.630	0.371	63.050	0.630	91	0.626	0.340	62.637	0.575
2		x																		10	100	92	0.598	0.406	59.783	0.596	91	0.571	0.431	57.143	0.579
3			x																	10	100	92	0.565	0.447	56.521	0.559	91	0.538	0.462	53.846	0.541
4				x																100	1	92	0.565	0.434	56.523	0.565	91	0.549	0.454	54.945	0.526
5					x															100	0.1	92	0.587	0.417	58.660	0.550	91	0.495	0.509	49.451	0.424
6						x														1	100	92	0.641	0.364	64.130	0.590	91	0.571	0.432	57.143	0.488
7							x													1	100	92	0.641	0.364	64.130	0.639	91	0.560	0.444	56.044	0.402
8								x												10	10	92	0.620	0.380	61.957	0.621	91	0.604	0.398	60.440	0.571
9	x						x													10	100	92	0.587	0.416	58.696	0.585	91	0.520	0.473	52.747	0.559
10	x					x	x													100	100	92	0.620	0.381	61.950	0.619	91	0.495	0.505	49.450	0.550
11	x	x	x	x	x	x	x													1	100	92	0.641	0.358	64.130	0.642	91	0.538	0.461	53.846	0.539
12	x	x	x	x	x	x	x	x												100	0.1	92	0.598	0.406	59.783	0.596	91	0.510	0.430	57.143	0.560
13											x		X							100	0.01	55	0.764	0.233	63.640	0.765	54	0.704	0.296	70.370	0.822
14												x								100	0.1	42	0.786	0.267	78.571	0.590	42	0.833	0.181	83.333	0.844
15	x								x											10	0.1	55	0.782	0.218	78.181	0.848	55	0.741	0.259	74.074	0.804
16									x	x	x	x	X	x						100	1	55	0.891	0.109	89.091	0.891	54	0.907	0.093	90.741	0.955
17	x								x	x	x	x	X	x						10	1	55	0.891	0.109	89.091	0.891	54	0.889	0.111	88.889	0.927
18	x	x	x	x	x	x	x	x	x	x	x	x	X	x						100	1	55	0.927	0.073	92.727	0.927	54	0.907	0.093	90.741	0.925
19															x	x	x	x	x	10	10	38	0.842	0.118	84.211	0.862	38	0.737	0.377	73.684	0.475
20															x					10	1	38	0.789	0.411	78.947	0.689	38	0.711	0.711	71.053	0.000
21																x				100	1	38	0.709	0.286	70.909	0.712	38	0.605	0.753	60.526	0.000
22																	x			10	1	38	0.789	0.411	78.947	0.689	38	0.684	0.721	68.421	0.175

*Hippo, hippocampus; LM, learning memory; COWA, controlled oral word association test (animals; and letters K, S, P); clock, clock drawing test; NPI4, depression; NPI5, anxiety; NPI7, apathy; NPI9, irritability; TP rate, true positive rate; FP rate, false positive rate; ROC, receiver operating characteristic; curve (stands for AUC—area under the curve—in this table). Blue = main flow; Yellow = subjects excluded; Green = action done to the data*.

However, from the results of the ROC analysis, the model consisted of TMSE, LM, COWA—animals, COWA-KSP, Clock drawing, and Construction—praxis scores provided the best performance in the diagnosis of AD with the AUC = 0.96 (Table 3, item 18). Other notable high performance models were found with these parameters: hippocampus with TMSE, LM, COWA—animals, COWA-KSP, clock drawing, and Construction—praxis (AUC = 0.927, Table 3, item 17). We also performed analyses on various combinations of features, but those revealed lower accuracies and lower AUC values as shown in Table 3.

## Discussion

Our study with SVM classification revealed that utilizing the scores of TMSE, LM, COWA—animals, COWA-KSP, Clock drawing, and Construction—praxis can best differentiate AD from normal controlled subjects. Neuropsychiatric symptoms alone could not provide accurate results. The demographic data in this study showed that the individuals in the AD group had a slightly older mean age (73.09 years) than those in the normal control group (69.72 years). Those with AD also had a lower formal educational level, as indicated by their comparatively smaller number of years of schooling. Previous research revealed that individuals with a lower number of years of formal education might have a smaller cognitive reserve. As a consequence, the incidence of AD has been reported to be higher in those individuals with lower education (16).

Previous studies showed that digital health data, cognitive performance such as memory, and neuropsychiatric symptoms can help identify those with dementia from normal subjects (17–21). Some research groups (19) have suggested that a diagnosis of dementia can be made from health recording data.

Despite using the highest accuracy that the SVM could provide, brain volumetry alone provided suboptimal accuracy for the diagnosis of AD. The highest accuracy for brain volumetry was found with the hippocampus region, which reached an accuracy of 62.64%. The inclusion of other regions of the brain did not seem to increase the accuracy level any further. This also reflects previous research findings that the hippocampus volume is associated with AD (22). Given that the mean baseline TMSE score of the AD group was not low, one possibility is that the SVM failed to classify AD accurately using hippocampal volume alone in our study because our AD subjects tended to have a mild-to-moderate degree of severity of the disease. Consequently, the difference in the hippocampal volumes of each group was not pronounced. Individuals in Asian counties are known to have a high prevalence of cerebral small vessel disease, which increases with age (23). This cerebral small vessel disease can cause smaller hippocampal volume in subjects with normal cognition. However, in a recent review of 111 studies, the majority of the studies assessed Alzheimer's disease compared with healthy controls, using AD Neuroimaging Initiative data, support vector machines, and only T1-weighted sequences (24). Accuracy was highest for differentiating Alzheimer's disease from healthy controls and poor for differentiating healthy controls vs. mild cognitive impairment vs. Alzheimer's disease.

Our study with SVM classification suggested that brain volumetry alone seems to be a suboptimal parameter for AD diagnosis; a different situation is found with the clinical parameters. COWA had a high degree of accuracy, especially COWA-KSP (note that KSP come from the letters “
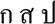
” in the Thai language; in English-speaking countries, the letters “F, A, and S” or “C, F, and L” are normally used). These results are consistent with the knowledge that individuals with AD also have executive function impairment of varying severities (25). Word fluency relies on the executive functions that will enable an individual to produce a number of words quickly in a limited time. It follows that COWA might be adapted as a good screening tool for the diagnosis of AD. A combination of other neuropsychiatric tests also afforded a very high degree of accuracy. However, utilizing neuropsychiatric symptoms to aid the diagnosis of AD resulted in poor ROC value in the tested data. This indicated that neuropsychiatric symptoms alone could not differentiate AD from norms or other dementia.

In the previous study, both structural T1-weighted MRI brain studies and neuropsychological measures of individuals were used to train and optimize an artificial intelligence classifier to diagnose mild-AD patients (26). Similar to our study, the classifier was able to distinguish between the two groups before AD definite diagnosis using a combination of MRI brain studies and specific neuropsychological measures, with 85% accuracy, 83% sensitivity, and 87% specificity.

## Limitations and Future Study

An important limitation was that the severity of the AD of our subjects seemed to be low to moderate. Although, the hippocampus alone was rather a good choice for use as a classification parameter, our study failed to demonstrate that it was. The low education of individuals in our study may play some role on brain volume. The second limitation was that this study did not utilize age-matched group due to the limited number of subject recruitments and the small number of subjects for the SVM training and testing groups. Generalization of the results to clinical use should be done with caution. The third limitation was that, we used dementia subjects from the memory clinic together with normal subjects from the community survey or community study, which could lead to selection bias. When applied to the broader general population, our method of using the SVM might not produce the same degree of accuracy.

Future studies should include a larger sample size in both the training and testing groups. It is known that the Asian population has a prevalence of small vessel disease in the brain. The burden of cerebral small vessel disease can be included to predict the diagnosis of dementia in the future. Exploration with other biomarkers to predict the diagnosis of Alzheimer's disease or prodromal Alzheimer's disease in a Thai cohort could also be done.

## Conclusions

Based on data drawn from the Memory Clinic at Siriraj Hospital, clinical parameters (including TMSE, COWA—animals, COWA-KSP, LM, clock drawing, and Construction—praxis) provided good accuracy (83–90%) using SVM as a classifier. COWA-KSP alone might be the easiest tool to utilize in clinical situations, and it had an accuracy of 83.33% when using the SVM. Our study failed to demonstrate a good degree of accuracy when using brain volumetry alone. The most accurate results were found using the hippocampus alone as a classifier that revealed an accuracy of 62.64%.

## Data Availability Statement

The original contributions presented in the study are included in the article/supplementary material, further inquiries can be directed to the corresponding author.

## Ethics Statement

The studies involving human participants were reviewed and approved by IRB Faculty of Medicine Siriraj Hospital. The patients/participants provided their written informed consent to participate in this study. Written informed consent was obtained from the individual(s) for the publication of any potentially identifiable images or data included in this article.

## Author Contributions

VS: research planning, primary investigators of the awarding grants, conducting the field study, data analysis and interpretation of the results, and preparing manuscript. YV and AK: assisted data analysis and interpretation of the results. AR, SC, and NA: conducted field study, assessment of cognition and neuropsychiatricproblems, and preparing the data for statistical analysis. All authors read and approved the final manuscript.

## Conflict of Interest

The authors declare that the research was conducted in the absence of any commercial or financial relationships that could be construed as a potential conflict of interest.
